# Enhancing therapeutic efficacy through degradation of endogenous extracellular matrix in primary breast tumor spheroids

**DOI:** 10.1111/febs.70069

**Published:** 2025-03-17

**Authors:** Alessandra Lo Cicero, Simona Campora, Gabriele Lo Buglio, Paolo Cinà, Margot Lo Pinto, Simone Dario Scilabra, Giulio Ghersi

**Affiliations:** ^1^ Department of Biological, Chemical and Pharmaceutical Sciences and Technologies (STEBICEF) University of Palermo Italy; ^2^ Department of Biomedical Engineering Bioscience Center of the University of Cincinnati OH USA; ^3^ Department of Pharmacy University of Copenhagen Denmark; ^4^ Abiel Srl Palermo Italy; ^5^ Proteomics Group of Ri.MED Foundation Research Department IRCCS ISMETT (Istituto Mediterraneo per i Trapianti e Terapie ad Alta Specializzazione) Palermo Italy

**Keywords:** breast cancer, collagen, collagenase, drug response, extracellular matrix, spheroids

## Abstract

Solid tumors have a complex extracellular matrix (ECM) that significantly affects tumor behavior and response to therapy. Understanding the ECM's role is crucial for advancing cancer research and treatment. This study established an *in vitro* model using primary cells isolated from a rat breast tumor to generate three‐dimensional spheroids. Monolayer cells and spheroid cultures exhibited different protein expression patterns, with primary tumor spheroids presenting an increased level of ECM‐related proteins and a more complex extracellular environment. Furthermore, spheroids produce endogenous collagen type I matrix, which is the main component of the tumoral ECM. This matrix is arranged predominantly around the 3D structure, mimicking the conditions of solid tumors. Treatments with recombinant collagenases class II (acting on the linear collagen region) and class I (acting on the 3D‐helix region) completely degrade collagen within the spheroid structure. Collagenase pretreatment enhances the accessibility of the anticancer drug doxorubicin to penetrate the core of spheroids and sensitize them to doxorubicin‐induced cytotoxicity. Our findings highlight the importance of overcoming drug resistance in breast cancer by targeting the ECM and proposing a novel strategy for improving therapeutic outcomes in solid tumors. By employing a three‐dimensional spheroid model, with an endogenous ECM, we can offer more relevant insights into tumor biology and treatment responses.

## Graphical abstract

Solid tumors have a complex extracellular matrix (ECM) that can significantly affect tumor behavior and treatment response. Understanding the role of the ECM is therefore critical for understanding tumor biology and improving therapeutic responses. In this study, we isolated primary cells from a DMBA‐induced tumor mass to generate three‐dimensional spheroids. Proteomic analysis showed that compared to the two‐dimensional monolayer cells, spheroids harbored a different expression of ECM components. Furthermore, treatment with recombinant collagenases completely digested the collagen matrix surrounding the spheroids, maintaining spheroid size while enhancing doxorubicin uptake and efficiency.

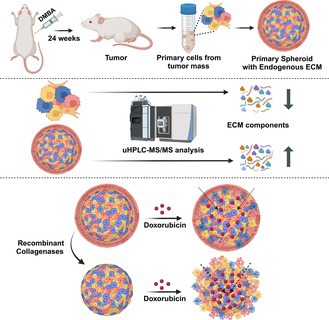

**Abbreviations**
CAF: cancer‐associated fibroblasts
DMBA: 7,12‐dimethylbenz(a)anthracene
ECM: Extracellular matrix
MMP: matrix metalloproteinase
PCTM: primary cells from the tumor mass
TME: tumor microenvironment

## Introduction

Cancer is a leading cause of death worldwide, with nearly 20 million new cases diagnosed and 10 million deaths reported in 2022 (World Health Organization), and the mortality rate is increasing every year. Solid tumors, which account for approximately 90% of adult human cancers, are characterized by their unique tumor microenvironment (TME). Over the last decades, to understand the complexity of solid tumor biology, research has focused more on the TME, a complex network that consists of tumor vasculature, associated connective tissues, infiltrating immune cells, and the extracellular matrix (ECM) [1, 2, 3]. The ECM, a dynamic structure undergoing continuous remodeling through synthesis and degradation mechanisms, plays a crucial role in various cellular signaling cascades involved in the promotion of tumor growth, invasion, and migration [4, 5, 6, 7, 8]. The ECM contains several fibrillar proteins, such as collagen, fibronectin, elastin, and laminin, mainly produced by cancer‐associated fibroblasts (CAF) [9]. Collagens are the primary components of the ECM, providing structural support to tissues and influencing cellular behavior through biochemical and mechanical mechanisms. Various types and isoforms of collagens are overexpressed in tumors and their organization and density can significantly impact tumor progression. For instance, collagen remodeling and crosslinking can enhance tumor stiffness, which is associated with increased tumor invasiveness and has a key role in drug resistance, acting as a physical barrier to therapeutic agents [10, 11]. Moreover, collagen can influence cellular signaling pathways that regulate the proliferation, survival, and migration of cancer cells.

Breast cancer, the most common cancer type among women, causes approximately half a million deaths annually worldwide (World Health Organization). The ECM is increasingly recognized as a significant regulator in breast cancer [12, 13, 14], exhibiting high levels of collagens organized in variable compositions indicative of different clinical outcomes [15, 16, 17, 18]. For instance, in breast cancer tissues, collagen type I, the most abundant collagen type in the human body and the primary component of the interstitial ECM, is significantly upregulated contributing to increased tissue stiffness and promoting tumor cell invasion and metastasis. Type III, involved in the early stages of breast cancer, and type XII collagen are also significantly overexpressed [19] and often correlated with poor outcomes [20].

The nature and composition of the ECM are hard to replicate *in vitro*, especially when working in a monolayer system. Three‐dimensional (3D) spheroids are cell aggregates that form complex networks of cell–cell contacts, features absent in traditional cell monolayers, thus better mimicking the *in vivo* system [21, 22]. Several studies have been conducted on 3D tumor culture systems, such as multicellular tumor spheroids [23, 24], on cell lines and primary cells that do not present an endogenous matrix and are cultured with coating techniques with ECM‐derived components [25, 26]. Due to their *in vivo*‐like composition, 3D spheroids serve as models for evaluating anticancer drug effectiveness and understanding the tumor mass's intrinsic properties [27]. Breast cancer cell lines have been successfully used to generate spheroids as promising models for medical application and drug discovery [28, 29, 30].

Thus, to develop a reliable model of a 3D spheroid with an endogenous ECM, we isolated primary cells from rat breast cancer to generate spheroids. Spheroids present an organized ECM, rich in collagen. By using recombinant collagenases to completely degrade the native collagen of the ECM in the 3D model, we demonstrated that ECM degradation can enhance drug efficacy by improving penetration and chemotherapeutic effects.

## Results

### Cell isolation from tumor mass and primary breast tumor spheroid formation

**Fig. 1:**
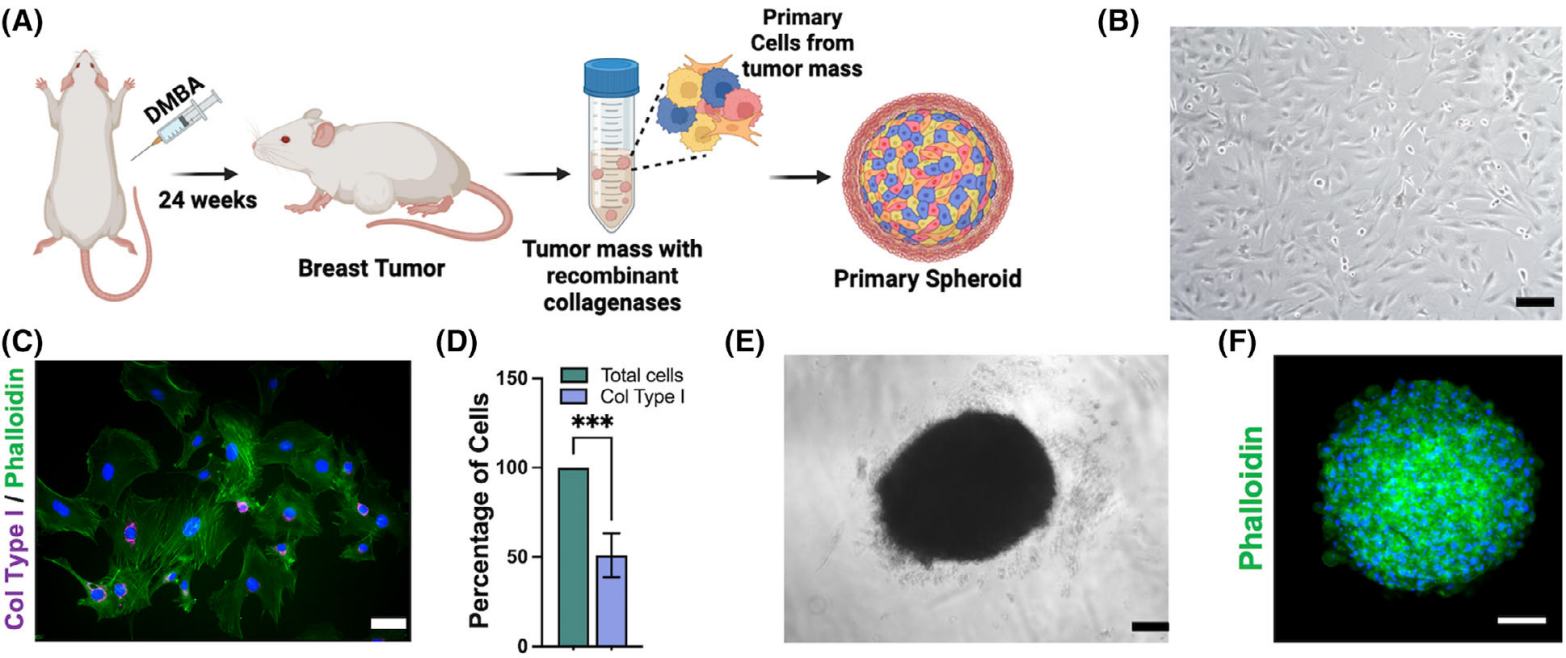
Cell isolation from tumor mass and primary breast tumor spheroid formation. (A) Schematic representation describing the induction of breast cancer with DMBA and tissue digestion to isolate cells and generate spheroids. (B) Primary cells isolated from the tumor mass (PCTM). (C) Widefield microscopy analysis of PCTM stained with phalloidin‐FITC (green), antibody anti‐collagen type I (magenta), and DAPI (blue) (D) and collagen type I‐positive cells. Scale bar: 15 μm. (E) 3D‐spheroid (F) and confocal microscopy analysis of the spheroids stained with phalloidin‐FITC (green) and DAPI (blue). Scale bar: 100 μm. Data are mean ± SD. Statistical analysis (*t*‐test) was performed (*n* = 3) (***P < 0.001).

To investigate the ECM in primary tumor spheroids, a breast tumor was induced in an immunocompetent female Wistar rat via intraperitoneal injection of a well‐established chemical carcinogen, 7,12‐dimethylbenz(a)anthracene (DMBA) (Fig. 1A) [31].

After 6 months, a tumor mass visible in the abdominal submammary region was excised and treated with recombinant collagenases and thermolysin to dissociate the tissue and isolate primary cells. The isolated primary cells from the tumor mass (PCTM) exhibited heterogeneous morphological characteristics, indicative of the diverse cellular composition of the tumor microenvironment (Fig. 1B). This heterogeneity was confirmed through collagen type I staining, which identified cells synthesizing collagen type I, the major ECM component (Fig. 1C,D).

To better mimic the tumor condition and functions, modulated by cell–cell and cell–ECM interactions, the isolated PCTM were seeded under low‐attachment conditions, promoting the formation of three‐dimensional (3D) spheroids. After 6 days in culture, the cells formed spheroids with a homogeneous morphology and consistent size, ranging from 400 to 450 μm in diameter (Fig. 1E,F).

### Proteomic analysis revealed an organized extracellular matrix in 3D primary breast tumor spheroids

**Fig. 2:**
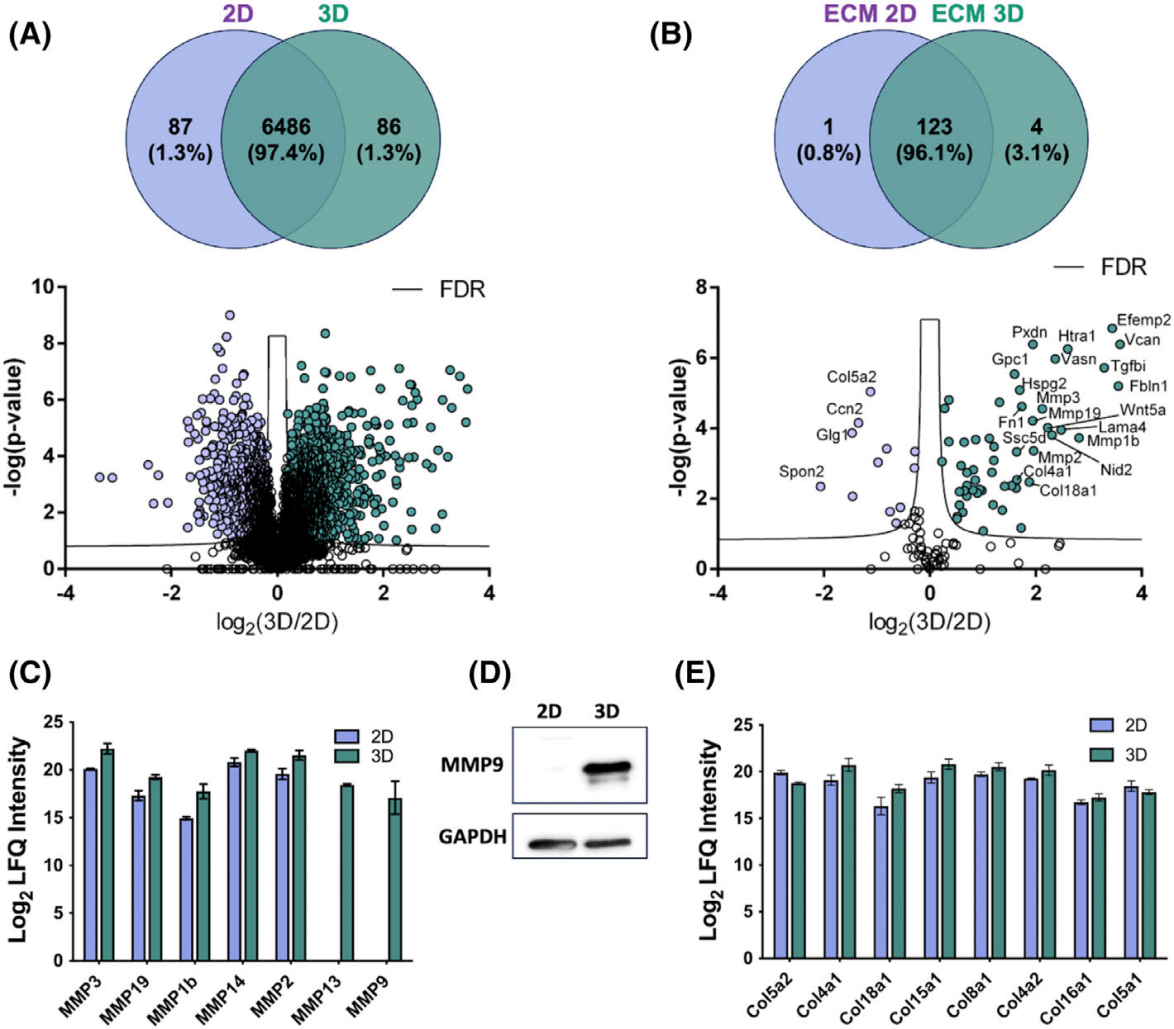
Proteomic analysis revealed an organized extracellular matrix in 3D primary breast tumor spheroids. (A) Venn diagram and Volcano plot for the proteins identified in five biological samples of the 2D cell culture and 3D spheroids; volcano plot showing the −log10 of *P*‐values versus the log2 of protein ratio for all proteins identified in the lysates of cells in 2D cell culture and 3D spheroids (*n* = 5). Blue: proteins of 3D spheroids; red: proteins of 2D cells. (B) Venn diagram and Volcano plot for the ECM proteins identified in five biological samples of the 2D cell culture and 3D spheroids; volcano plot showing the −log10 of *P*‐values versus the log2 of protein ratio for ECM proteins identified in the lysates of cells in 2D cell culture versus 3D spheroids (*n* = 5). Blue: proteins of 3D spheroids; red: proteins of 2D cells. (C) LFQ (label‐free quantification) intensity of the most significant MMPs (Matrix Metalloproteinases) differently expressed in 2D and 3D cultures (*n* = 5). (D) Western blot analysis of total cell lysates (15 μg) from 2D cell cultures (2D) and 3D spheroids (3D), using anti‐MMP9 antibody. (E) LFQ intensity of the most significant types of collagen present in 2D and 3D cultures (*n* = 5). Data are mean ± SD.

3D spheroids and two‐dimensional (2D) primary cells were analyzed through high‐resolution proteomics to identify differential protein abundance in 2D and 3D systems, focusing on proteins involved in ECM organization. The analysis identified 6572 proteins in PCTM spheroids and 6573 proteins in cells grown in 2D, while 6486 proteins were common to both spheroids and 2D cells (Fig. 2A, Table S1). Among these common proteins, 1128 proteins were more abundant in the 3D spheroids, whereas 816 proteins were less abundant compared to the 2D cell culture (Table S1). This differential expression highlights the distinct proteomic landscapes of 2D and 3D systems, reflecting their different microenvironments and cellular interactions. To further explore the distinctive organization of the ECM, we focused on changes in ECM proteins (according to the UniProt Gene Ontology Cellular Component 0031012). This analysis identified 128 ECM‐related proteins, with 12 proteins significantly decreased in PCTM spheroids (Fig. 2B, Table 1 and Table S2). Conversely, 45 proteins were significantly augmented in primary tumor spheroids (Fig. 2B, Table 2 and Table S2). The increased levels of these proteins in 3D spheroids indicate enhanced ECM remodeling and a more complex extracellular environment, which is more representative of tumor conditions. Notably, the expression of matrix metalloproteinases (MMPs), such as MMP2, MMP19, MMP1b, MMP14, and MMP3, increased in the 3D system, suggesting enhanced ECM remodeling (Fig. 2C). Interestingly, MMP9 and MMP13 were exclusively present in spheroids, pointing to unique proteolytic activities in the 3D context (Fig. 2D). These findings are significant as MMPs play crucial roles in tumor invasion and metastasis. Additionally, various types of collagens, such as Col5a2, Col4a1, Col18a1, Col5a1, Col8a1, Col4a2, Col16a1, and Col5a1, were identified in both 2D and 3D systems. However, these collagens were upregulated in 3D spheroids (Fig. 2E), indicating a more extensive and structurally complex ECM in the 3D model.

**Table 1:** List of the 12 most highly reduced ECM proteins in 3D spheroids compared to 2D cell cultures. Protein name: name of ECM proteins reduced in primary tumor spheroids. Protein ID: UniProt accession number of the protein. Gene name: UniProt gene name associated with each protein. *P‐value*: *P*‐value calculated after a Student *t*‐test of five biological replicates. Ratio: ratio between the mean of LFQ values of primary tumor spheroids and tumor cells in 2D cultures (*n* = 5).

Protein name	Protein ID	Gene name	*P*‐value	Ratio
Spondin‐2	Q9WV75	Spon2	4.46E‐03	0.23873
Golgi apparatus protein 1	Q62638	Glg1	1.34E‐04	0.36147
Laminin subunit gamma 2	F1LRH4	Lamc2	8.48E‐03	0.36393
CCN family member 2	Q9R1E9	Ccn2	7.02E‐05	0.39299
Collagen type V alpha 2 chain	F1LQ00	Col5a2	9.06E‐06	0.45915
Integrin subunit alpha 6	G3V667	Itga6	9.18E‐04	0.50645
Tissue factor	P42533	F3	3.78E‐04	0.56778
Lumican	P51886	Lum	2.32E‐02	0.59290
Collagen alpha‐1(V) chain	Q9JI03	Col5a1	4.91E‐02	0.64259
Extracellular superoxide dismutase [Cu‐Zn]	Q08420	Sod3	1.76E‐02	0.67894
Prolyl 3‐hydroxylase 1	Q9R1J8	P3h1	1.32E‐03	0.81622
Metalloproteinase inhibitor 1	P30120	Timp1	4.52E‐04	0.82295

**Table 2:** List of the 23 most highly increased ECM proteins increased in 3D spheroids compared to 2D cell cultures. Protein name: name of ECM proteins increased in primary tumor spheroids with a 3D/2D ratio above 2.82 (which is 1.5 in a log2 scale). Protein ID: UniProt accession number of the protein. Gene name: UniProt gene name associated with each protein. *P‐value*: *P*‐value calculated after a Student *t*‐test of five biological replicates. Ratio: ratio between the mean of LFQ values of primary tumor spheroids and tumor cells in 2D cultures (*n* = 5).

Protein name	Protein ID	Gene name	p‐value	Ratio
Versican core protein	Q9ERB4	Vcan	4.06E‐07	12 040
Fibulin‐1	A0A0G2JY81	Fbln1	6.23E‐06	11 779
EGF containing fibulin extracellular matrix protein 2	A0A8I5Y8F2	Efemp2	1.44E‐07	10 869
Transforming growth factor, beta induced	D4A8G5	Tgfbi	1.88E‐06	9794
Matrix metallopeptidase 1b (interstitial collagenase)	D3ZRZ2	Mmp1b	1.86E‐04	7042
Serine protease HTRA1	Q9QZK5	Htra1	5.55E‐07	6081
Laminin subunit alpha 4	F1LTF8	Lama4	1.10E‐04	5574
Vasorin	D3ZAE6	Vasn	1.06E‐06	5158
Nidogen‐2	B5DFC9	Nid2	1.55E‐04	4942
Protein Wnt‐5a	Q9QXQ7; B1WBR9	Wnt5a	9.47E‐05	4673
Stromelysin‐1	P03957	Mmp3	2.79E‐05	4357
72 kDa type IV collagenase	P33436	Mmp2	4.31E‐04	3884
Peroxidasin	A0A0G2JWB6	Pxdn	4.05E‐07	3845
Matrix metallopeptidase 19	C0M4B0	Mmp19	6.11E‐05	3836
Collagen type XVIII alpha 1 chain	A0A8I6GLR1	Col18a1	3.36E‐03	3677
Fibronectin	P04937	Fn1	2.37E‐05	3326
Fibulin‐5	Q9WVH8	Fbln5	6.73E‐02	3298
Heparan sulfate proteoglycan 2	A0A8I6ASH2	Hspg2	8.15E‐06	3236
Collagen type IV alpha 1 chain	F1MA59; A0A8I5ZZ86	Col4a1	2.88E‐03	3121
Scavenger receptor cysteine rich family member with 5 domains	D3ZPK4	Ssc5d	4.60E‐04	3117
Apolipoprotein E	P02650	Apoe	4.94E‐03	3092
Glypican‐1	P35053	Gpc1	2.87E‐06	3025
Glia‐derived nexin	P07092	Serpine2	4.30E‐03	2909

### Primary breast tumor spheroids produce an endogenous extracellular matrix, which is completely degraded with recombinant collagenases

Since proteomic analysis revealed a different collagen expression between 2D and 3D systems, collagen type I, the most abundant collagen in tumoral ECM, was further investigated by confocal microscopy to evaluate the organization and distribution within and around the spheroids.

**Fig. 3:**
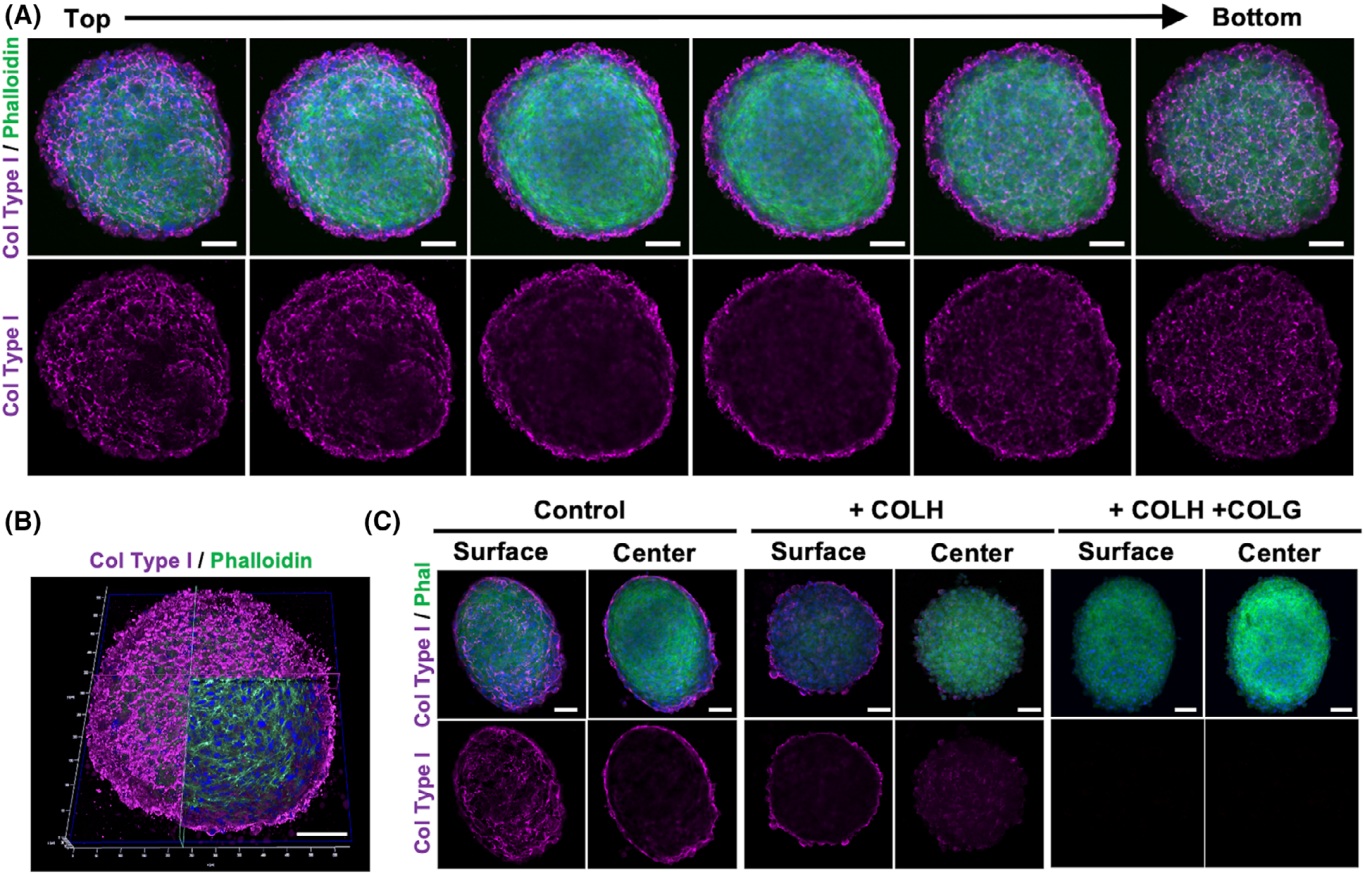
Primary breast tumor spheroids produce an endogenous extracellular matrix, which is completely degraded with recombinant collagenases. (A) Confocal microscope stacks from top to bottom of primary tumor spheroids, stained with phalloidin‐FITC (green), antibody anti‐collagen type I (magenta), and DAPI (blue). (B) A section at the confocal microscope. Scale bar: 100 μm. (C) Primary tumor spheroids untreated (control), treated by recombinant collagenase COLH (+COLH) for 1 h 15 min, and treated by recombinant collagenases COLG and COLH (+COLH/COLG) for 1 h 15 min. After the enzymatic treatment, spheroids were stained with phalloidin‐FITC (Phal) (green), antibody anti‐collagen type I (magenta), and DAPI (blue) and analyzed with confocal imaging, showing the surface (left) and the center (right) of spheroids. Scale bar: 100 μm. Experiments have been conducted three times, ‘*n* = 3’.

Immunostaining highlighted the capability of primary spheroids to produce an endogenous ECM, rich in collagen, which is mainly arranged around the 3D structure, simulating *in vitro* the tumor microenvironment conditions in solid tumors (Fig. 3A). To evaluate the effectiveness of collagenases in digesting collagen expressed in primary tumor spheroids, experiments were conducted by incubating spheroids with either ultrapure recombinant COLH (collagenase class II, acting on the linear collagen region) or COLH in combination with COLG (collagenase class I, acting on the 3D‐helix region) (Fig. 3B). Confocal microscopy observation revealed a reduction in type I collagen in spheroids incubated with COLH alone. However, complete digestion of type I collagen was observed when COLH was combined with COLG, demonstrating the synergistic effect of these enzymes in degrading collagen within the spheroid structure (Fig. 3C). The complete digestion indicates the effective breakdown of the collagen matrix, essential for subsequent experimental manipulations and assessing the impact of ECM degradation on drug delivery.

### Collagenase treatment improves doxorubicin uptake and cytotoxicity effects in primary breast tumor spheroids

**Fig. 4:**
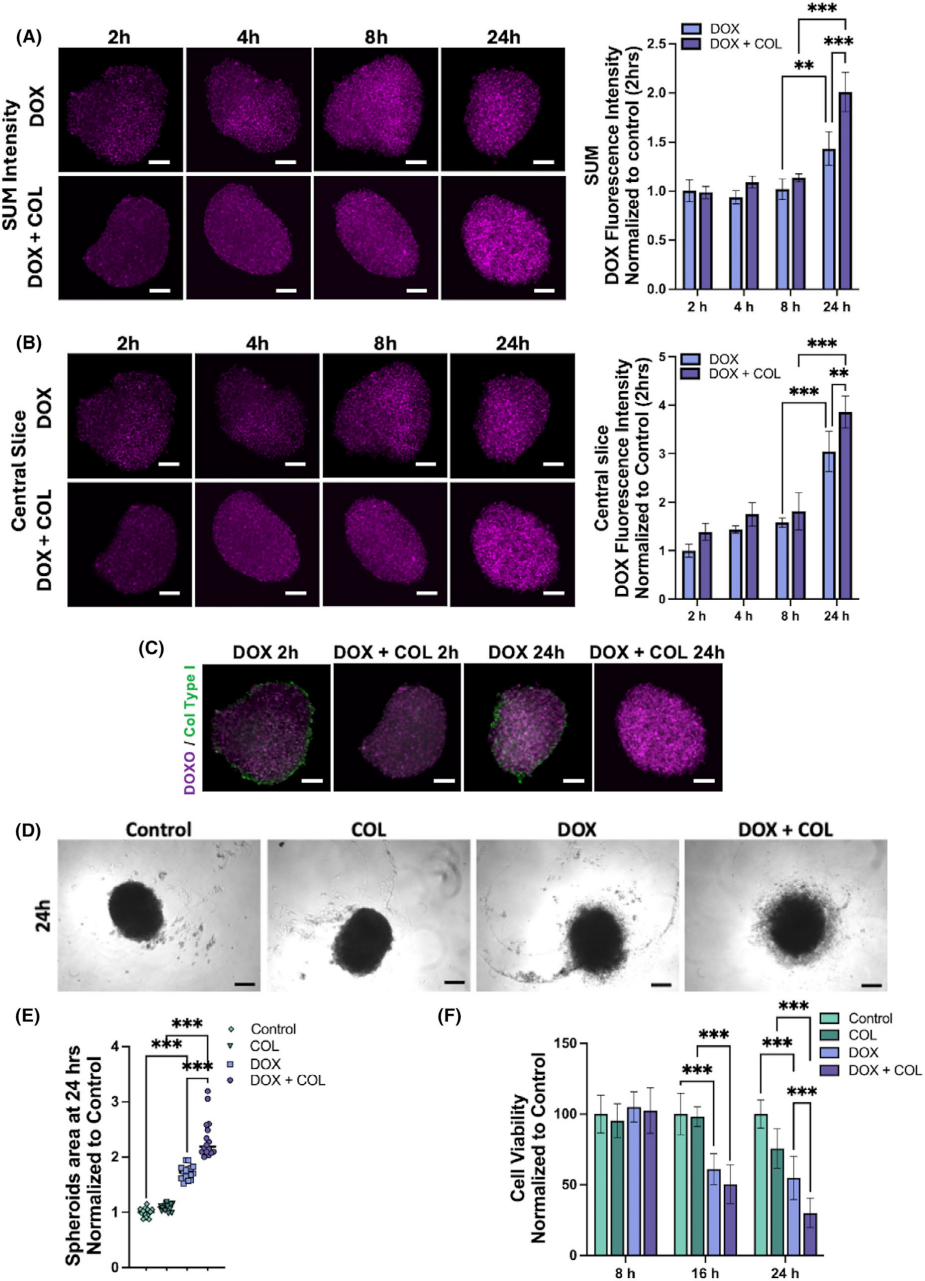
Collagenase treatment improves doxorubicin uptake and cytotoxicity effects in primary breast tumor spheroids. (A) Confocal analysis (stacks sum intensity) of 20 μm doxorubicin uptake in control (DOX) spheroid and collagenase‐treated (DOX + COL) at different time points (2, 4, 8, 24 h). Quantification of the fluorescence Sum intensities was analyzed and normalized to control. (B) Confocal analysis (central stack intensity) of 20 μm doxorubicin uptake in control spheroid and collagenase‐treated at different time points (2, 4, 8, 24 h). Quantification of the central stack fluorescence intensities was analyzed and normalized to control. Magenta: DOX autofluorescence. Scale bar: 100 μm. Statistical analysis (two‐way ANOVA) was performed (*n* = 3). (C) Confocal analysis (stacks sum intensity) of 20 μm doxorubicin uptake in control (DOX) spheroid and collagenase‐treated (DOX + COL) at different time points (2 and 24 h). Magenta: DOX autofluorescence, Green: anti‐collagen type I. Scale bar: 100 μm. (D) Representative pictures of control primary tumor spheroid (Control), treated with collagenases (COL), treated with doxorubicin 20 μm (DOX) and with collagenases + doxorubicin 20 μm (DOX + COL) at 24 h (scale bar: 10 μm). (E) Quantification of primary tumor spheroids area at 24 h, including dead cells, normalized to the area at T0 (not shown in this figure). Statistical analysis (one‐way ANOVA) was performed (*n* = 3). (F) Cell viability assay of primary tumor spheroid, treated with collagenases (COL), treated with doxorubicin 20 μm (DOX) and with collagenases + doxorubicin 20 μm (DOX + COL) at different times (8, 16, 24 h). Scale bar: 100 μm. Data are mean ± SD. Statistical analysis (two‐way ANOVA) was performed (*n* = 3) (**P < 0.01, ***P < 0.001).

To investigate the impact of ECM degradation on the accessibility into a 3D model of the commonly used anticancer drug doxorubicin, uptake assays were carried out on PCTM spheroids. Spheroids were incubated with doxorubicin after 6 days of formation, and the drug's uptake was monitored over time. A significant increase in the doxorubicin signal was evident both in the entire spheroids (Fig. 4A) and their central slice (Fig. 4B) in a time‐dependent manner. Maximum intensity was observed at 24 h of incubation, whereas in PCTM 2D cell culture, maximum doxorubicin uptake and nuclear translocation were detectable after only 2 h (Fig. S1). This slower uptake in spheroids highlights the barrier effect of the ECM on drug penetration. Furthermore, analysis of the core of the spheroids underlined that doxorubicin uptake increased significantly over time relative to the total intensity, suggesting that the drug initially accumulates in the outer layer of the spheroids and then penetrates towards the core by 24 h (Fig. 4B). Based on the optimized concentration of recombinant collagenases (COLG and COLH), PCTM spheroids were predigested with collagenases before doxorubicin treatments (Fig. 4C). At 24‐h post‐treatments, these spheroids displayed a 38% increase in doxorubicin uptake signal in the total intensity (Fig. 4A) and a 26% increase in the central slice (Fig. 4B), compared to spheroids treated with doxorubicin alone. This enhanced uptake post‐collagenase treatment underscores the significance of ECM degradation in improving drug penetration and efficacy within 3D tumor models.

To evaluate the impact of collagen degradation on the cytotoxic effects of doxorubicin in PCTM spheroids, they were first digested with collagenases, followed by treatment with doxorubicin for 24 h (Fig. 4D). After the treatment, the total area of spheroids, including areas of dead cells, was analyzed. Interestingly, spheroids treated with collagenases alone did not exhibit a significant change in size compared to the untreated control, indicating that collagenase treatment by itself did not induce notable cytotoxic effects. In contrast, treatment with doxorubicin alone resulted in a 75% increase in the size of spheroids. When the spheroids' collagen matrix was predigested with collagenases before doxorubicin treatments, the drug's effects were significantly enhanced. This combination led to a 34% increase in doxorubicin efficacy compared to spheroids treated with doxorubicin alone (Fig. 4E).

The role of collagenases in enhancing drug efficacy was further investigated by analyzing the doxorubicin cytotoxicity effect in PCTM spheroids over time, with and without prior collagenase treatment. After 8 h of treatments, no significant cytotoxic effects were observed, indicating that short‐term exposure was insufficient to induce noticeable cell death. However, after 16 h, cytotoxic effects became more evident, although there was no significant difference between the two conditions at this stage. By 24 h, the collagenase treatments induced a slow decrease in cell viability, probably due to the release of collagen's peptides with inhibitory effects on cell proliferation. However, the cytotoxic effects of doxorubicin were more pronounced. Spheroids that had been pretreated with collagenases showed a 50% decrease in cell viability compared to those treated directly with doxorubicin (Fig. 4F). These findings reinforce the observation that ECM degradation enhances drug efficacy, as previously indicated by the increased drug uptake and changes in the spheroid area.

## Discussion

Tumorigenesis is a complex and dynamic process in which the ECM plays a leading role. In solid tumors, a stiff ECM and its remodeling are involved in different mechanisms in tumor progression, metastasis, immune escape [32], and in resistance to established cancer therapies [6, 8, 33].

Therefore, novel antitumor approaches should target cancer cells and dysregulated tumor ECM, especially fibrillar collagens that are the most altered in tumor ECM [34, 35].

The increasing focus on the tumor microenvironment presents a new challenge to obtain 3D reliable *in vitro* models for preclinical drug studies, which mimic an ECM similar to the tumor mass [36, 37]. In this context, many studies have reported the generation of 3D spheroid models using various cell lines [38, 39]. Furthermore, the involvement of primary cells, as proposed, can improve the reliability of the system. For this purpose, PCTM spheroids were generated using cells isolated from a chemically induced tumor mass in Wistar rats. PCTMs are a mixture of heterogeneous primary cells that self‐assembled to form uniform, round, compacted spheroids of consistent size (400–450 μm), with minimal variability in size and shape. Additionally, they are able to produce a well‐organized, endogenous collagen matrix with a capsule‐like structure, limiting the accessibility of drugs, as observed in solid tumors. Consequently, it is unnecessary to add additional ECM, as embedding them in a collagen gel, as is commonly reported [39, 40]. The proteomic analysis comparing cells grown in 2D to those in the 3D system revealed differences in protein expression and an enhanced ECM organization in the 3D system. Notably, tumor spheroids showed increased expression of proteins associated with solid tumor progression, such as versican and MMPs. In cancer, versican, a member of the proteoglycan family, impacts proliferation, survival, invasion, metastasis, and inflammation [41, 42]. MMP9 and MMP13 were specifically expressed in the 3D system and are well known for their involvement in ECM regulation, promoting cell proliferation and extravasation [43, 44, 45, 46]. Different types of collagens were also upregulated and this upregulation could influence cell behavior, signaling pathways, and drug response, making 3D spheroids a more relevant model for studying tumor biology and testing therapeutic interventions. Collagen plays a complex role in tumors, depending on the tumor origin, and different collagen types are closely associated with cancer progression and poor prognosis [18, 47, 48]. Therefore, collagen is considered a potential target for cancer treatment, and breaking it down could facilitate the penetration of chemotherapeutic agents. While collagenases are already used clinically for debridement in wounds and ulcers, their use in cancer treatments has not been validated yet [10, 49]. In this study, the digestion of spheroids' collagen was optimized using recombinant collagenases by combining two classes of these enzymes: Class I and Class II. Class I (COLG) recognizes the native collagen, acting on the 3D‐helix region, while Class II (COLH) has a higher activity against the linear collagen region [50]. The use of ultrapure recombinant collagenases significantly limits the side effects associated with proteases present in extracted collagenases, which can partially damage spheroids and cause the cells' escape from the spheroid structure. For the first time, this collagen digestion method enables the complete removal of the collagen matrix without altering the spheroid shape. This aspect is fundamental for *in vitro* models, as it allows for the selective study of collagen's role in drug response and the metabolic pathways of cells within a solid tumor mass.

The combination of COLG and COLH on primary tumor spheroids exhibits synergistic activity, resulting in efficient collagen digestion. Furthermore, to investigate the role of ECM on drugs' effects, the time‐dependent penetration of doxorubicin into the spheroid core was analyzed. The data emphasize the need for prolonged drug exposure in 3D cultures to achieve effective treatment, contrasting with the rapid uptake observed in 2D cultures. Moreover, the collagenase digestion experiments demonstrated that effective ECM degradation facilitates deeper penetration of therapeutic agents like doxorubicin, which is otherwise hindered by the dense ECM network. The enzymatic treatments enhanced the cytotoxicity effects of doxorubicin, suggesting potential therapeutic strategies that combine ECM degradation with chemotherapy to improve treatment outcomes.

These findings highlighted a novel model of primary tumor spheroids with an endogenous ECM, providing a more physiologically relevant platform for studying tumor biology and evaluating potential treatments. Moreover, this study provides critical insights into the role of the ECM in tumor spheroid models and its impact on drug delivery. Collagenase's proteolytic activity could improve not only the permeability of tumors to chemotherapeutic agents but also potentially facilitate the infiltration of immune cells into the mass. It is well established that the number of T cells found within a tumor as well as their ability to migrate and reach cancer cells is fundamental for an effective antitumoral response. However, the increased stiffness of ECM produced by tumors can directly regulate T‐cell migration [51], acting as a physical barrier and consequently impairing the infiltration of immune cells in the tumor mass [8, 52, 53].

In addition, recombinant collagenases could be a viable strategy to improve the delivery and efficacy of chemotherapeutic agents in solid tumors, potentially overcoming one of the significant obstacles in cancer treatment. Further research and *in vivo* studies are necessary to validate these findings and determine the safety, optimal dosing, and long‐term benefits of using collagenases in combination with chemotherapeutic agents in cancer therapy.

## Materials and methods

### Rat model

All animal experiments were approved by the University of Palermo. Authorization number 523/2022‐PR, issued according to art. 31 del D.lgs. 26/2014 Istituto Superiore di Sanità, Italy. The animals were housed 2 per cage (with EPA filter; controlled temperature 23 °C ± 0.5 °C; controlled humidity 50% ± 5%). The dark/light cycle was set at 12 h (7 a.m. to 7 p.m. light; 7 p.m. to 7 a.m. dark). Access to food at libitum.

Female Wistar rats were obtained from ENVIGO RMS SRL. At 55 days of age, a single dose of 50 mg·kg^−1^ 7,12 dimethylbenz(a)anthracene (DMBA) resuspended in corn oil was intraperitoneally injected [31]. After 6 months, an evident breast tumor mass was individuated.

The rat with a visible tumor mass was sedated with 2% isoflurane, sacrificed, and the tumor excised. A portion of the tumor was processed to isolate cells.

### Primary cells

Primary cells were isolated from DMBA‐induced tumors in female Wistar rats. The growth media used was Dulbecco's modified Eagle's medium (DMEM, Sigma‐Aldrich, Milano, Italy) high glucose (HG‐DMEM), which was supplemented with 10% fetal bovine serum (FBS), 100 U·mL^−1^ penicillin, 100 mg·mL^−1^ streptomycin (Euroclone, Celbar, Pero, Italy), and 2 mm l‐Glutamine (Euroclone, Celbar). The cells were then incubated at 37 °C, in an incubator at 5% CO_2_. Cell culture media was replenished every 3 days.

### Primary cancer cell isolation

Primary cells were isolated from DMBA‐induced tumors in female Wistar rats. The animal was sedated with 2% isoflurane, sacrificed and the tumor mass was explanted, washed in 70% ethanol, and put in Dulbecco's modified Eagle's medium (DMEM, Sigma‐Aldrich, Milano, Italy) high glucose (HG‐DMEM) added with 300 U·mL^−1^ penicillin, 300 U·mL^−1^, streptomycin (Euroclone, Celbar). After dissection in small parts with a scalpel, the tissue was incubated with the digestion buffer, containing ultrapure recombinant collagenases class I (Col G 106.4 U·g^−1^) and class II (Col H 453.2 U·g^−1^) (Abiel) and Thermolysin (Promega, Madison, Wisconsin, US ) 266.4 μg·g^−1^. The digestion was performed at 30 °C for 2 h in agitation and then stopped with HG‐DMEM containing 10% (v/v) fetal bovine serum (FBS, Euroclone, Celbar). The sample was washed three times with DMEM and centrifuged at 500 ***g*** for 5 min to isolate cells. The pellet was resuspended in DMEM/F12 medium added with 300 units·mL^−1^ penicillin G 300 mg·mL^−1^, streptomycin (Euroclone, Celbar), 2 mm l‐Glutamine (Euroclone, Celbar) and 10% (v/v) FBS and seeded on 50 μg·mL^−1^ (in 0.02 N acetic acid) collagen‐coated dishes and cultivated at 37 °C, in a humidified atmosphere of 5% CO_2_ in sterile conditions [54].

### Cells and spheroid culture

Primary isolated cells were cultured in DMEM high glucose plus 1% penicillin/streptomycin, 1% l‐Glutamine, and 10% FBS for 15 passages. Cells were maintained at 37 °C in a humidified atmosphere with 5% CO_2_. To form spheroids, primary cancer cells were seeded into a 96‐multiwell low‐attachment plate coated with agarose, at 1 × 10^4^ cells per well, in complete DMEM, at 37 °C and 5% CO_2_ and were let grow for 6 days.

### Proteomic‐based characterization: protein extraction and sample preparation

Cells from two‐dimensional and three‐dimensional cultures were lysed in STET (10 mm Tris/HCl, 1 mm EDTA, 100 mm NaCl, and 5% Triton X‐100 (v/v)) with a protease inhibitor cocktail (Roche, Basel, Switzerland) and centrifuged (14,000 x ***g***, 5 min) to eliminate cell membranes. Proteins extracted were quantified with a colorimetric 660 nm micro BCA assay. A protein amount of 30 μg per sample was subjected to filter‐aided sample preparation (FASP—with 10 kDa Vivacon 500 spin filter columns from Sartorius, Gottinga, Germany), as previously described [55]. Samples were then reduced and denatured with dithiothreitol (DTT) (Sigma‐Aldrich) in 200 μL of UA buffer (8 m Urea in 0.1 m Tris/HCl, pH 8.5) and alkylated with 50 mm iodoacetamide (IAA) (Sigma‐Aldrich). After reduction and alkylation, samples were washed three times with UB buffer (8 m Urea in 0.1 Tris/HCl, pH 8.0) and sequentially digested with LysC (1 : 50 enzyme to protein ratio from Promega) and trypsin (1 : 100 enzyme to protein ratio, Promega). Then, peptides were eluted from filter columns by centrifugation (14 000 ***g***; 60 min) and acidified with 20 μL of 8% formic acid (FA). Generated peptides were desalted by stop‐and‐go extraction (STAGE) on reverse phase C18 and eluted in 40 μL of 60% acetonitrile in 0.1% formic acid [56]. The volume was reduced in a SpeedVac (Thermo Fisher Scientific, Waltham, Massachusetts, US), and the peptides were resuspended in 20 μL of 0.1% formic acid. The peptide concentration was measured with NanoDrop 2000 (Thermo Scientific).

### uHPLC–MS/MS and data analysis

As previously described [57] for each sample, 1 μg peptides were separated through a nanoLC system (Vanquish Neo UHPLC, Thermo Scientific) equipped with an Acclaim PEPMap C18 column (25 cm × 75 μm ID, Thermo Scientific) in a 130 min binary gradient of water and acetonitrile containing 0.1% FA. Separated peptides were injected into an Exploris 480 mass spectrometer (Thermo Fischer Scientific) for tandem mass spectrometry analysis. Proteins were identified and quantified with label‐free quantification (LFQ) by using data‐independent acquisition (DIA). DIA was performed using an MS1 full scan (400–1200 m/z) followed by 60 sequential DIA windows with an overlap of 1 m/z and window placement optimization option enabled. Full scans were acquired with 120 000 resolution, AGC of 3 × 10^6^, and a maximum injection time of 50 ms. Afterward, 60 isolation windows were scanned with a resolution of 30 000, an AGC of 8 × 10^5^ and maximum injection time was set as auto to achieve the optimal cycle time. Collision‐induced dissociation fragmentation was induced with 30% of the normalized HCD. The data were analyzed by the software dia‐nn (version 1.8.1) by using a predicted library generated from *in silico* digested Rattus norvegicus (rat) UniProt reference database (proteome ID UP000002494) involving cuts at K* and R*, two missed cleavages allowed, minimal peptide length set a 6. The false discovery rate for peptide and protein identification was set at 0.01%. Label‐free quantification (LFQ) was used for protein quantification. The LFQ values were log2 transformed, and a two‐sided Student's *t*‐test was used to evaluate proteins statistically significantly regulated between two‐dimensional and three‐dimensional culture (*n* = 5). The mass spectrometry proteomics data have been deposited to the ProteomeXchange Consortium via the PRIDE partner repository with the dataset identifier PXD061082.

### Western blot analysis

Cells (2D and 3D) were lysed on ice in RIPA buffer (20 mm Tris, 150 mm NaCl, 0.1% Triton X‐100, 1 mm EDTA, pH 7.2) with a protease inhibitor cocktail. Lysates were incubated in a sample buffer under reducing conditions and fractionated with Sodium dodecyl sulfate–polyacrylamide gel electrophoresis (SDS/PAGE) 10% and transferred to nitrocellulose membranes (Amersham). The membranes were blocked in PBS/Tween 0.1% (PBS/T) with 5% non‐fat dried milk, incubated with the MMP‐9 (Abcam, Cambridge, UK, ab38906) primary antibody diluted 1 : 1000 in PBS/T, washed four times in blocking solution, and incubated with HRP‐conjugated secondary antibodies (Sigma) followed by washing in PBS/T.

The membrane was exposed to Super Signal West Femto Maximum Sensitivity substrate (Thermo Scientific), and protein expression was detected by chemidoc xrs (Bio‐Rad, Hercules, CA, USA).

### Immunofluorescence staining and imaging

Cells were fixed in 3.7% formaldehyde (1 h for spheroids, 15 min for 2D cells, at room temperature) before permeabilization in PBS supplemented with 0.1% Triton X‐100 (5 min, room temperature, only for the 2D cells). They were then blocked for 1 h at room temperature using PBS with 1% BSA (Sigma‐Aldrich, St. Louis, MO, USA). The primary antibody used was Rabbit anti‐collagen type I (1 : 20, 234167, Millipore, Burlington, Massachusetts, US), which was incubated overnight at 4 °C in a blocking buffer. Cells were stained with the species‐specific fluorophore‐conjugated secondary antibody (Invitrogen, Waltham, Massachusetts, US) (2 h for spheroids, 1 h for 2D cells, at room temperature), actin cytoskeleton was labeled with phalloidin‐FITC (1 : 500 in PBS) for 30 min at room temperature, and nuclei were visualized with DAPI (1 : 20 000 in PBS). Coverslips were mounted in a mounting medium and examined on the confocal microscope (Olympus FV10i and Stellaris 5, Leica, Wetzlar, Germany). Z‐stack imaging was carried out using a Fluoview FV10i confocal laser scanning microscope system (Olympus, Tokyo, Japan) at a magnification of 20× with a step size of 10 μm. All stacks were obtained at the same intensity setting between groups. imagej software (NIH, US) was used to analyze stacks of images.

### Collagenase treatment of spheroids

Spheroids were grown for 6 days, as described, washed two times in PBS, and then incubated with recombinant collagenases (Abiel srl) (20 U·mL^−1^ COLG and 80 U·mL^−1^ COLH) in DMEM without FBS for 1 h 15 min at 37 °C. After incubation, they were washed two times with DMEM with FBS to block collagenase activity.

### Doxorubicin uptake study by confocal microscopy

To assess the uptake of doxorubicin, spheroids untreated and treated with recombinant collagenases were incubated with 20 μm doxorubicin for 2, 4, 8, or 24 h. Spheroids were then processed for immunocytochemistry, as previously described. imagej software was used to analyze stacks of images.

### Spheroids' area analysis

Spheroids, untreated or treated with collagenases, were incubated with 20 μm doxorubicin for 24 h at 37 °C. After treatment, spheroids' areas were analyzed, including areas of dead cells. The analysis was performed with imagej.

### Cell viability assay

Spheroids, untreated or treated with collagenases, were incubated with 20 μm doxorubicin for 8, 16, and 24 h at 37 °C. After treatment, spheroids were washed three times with PBS, resuspended in 50 μL of DMEM and an equal volume of CellTiter‐Glo® 3D Reagent (Promega) following the Manufacturer's protocol. Briefly, 100 mL medium was replaced with 100 mL CellTiter‐Glo 3D reagent on the indicated day after growth/treatment. The plates were shaken for 5 min at 240 ***g***, incubated for 25 min at room temperature, and the luminescent signal was recorded using a luminometer (SinergyHT, BioTek, Winooski, VT, USA).

### Statistical analysis

Statistical analyses were performed using graphpad prism software (version 8.4.3) (Boston, MA, US). One‐way ANOVA with Tukey's *post hoc* test and two‐way ANOVA were used. The results are displayed as Mean ± SD, with significance at a level of *P*‐value < 0.05. Three to five replicates were analyzed for each experimental group. The experiments were performed three times.

## Conflict of interest

The authors declare no conflict of interest.

## Author contributions

ALC, SC, and GG designed the experiments. ALC, SC, and GLB performed the experiments. PC produced the recombinant collagenases. MLP and SDS performed the proteomic analysis. SC and GLB helped with data analysis. ALC analyzed data and wrote the manuscript. All authors reviewed and edited the manuscript and approved the final version of the manuscript.

## Peer review

The peer review history for this article is available at https://www.webofscience.com/api/gateway/wos/peer‐review/10.1111/febs.70069.

## Supporting information


**Fig. S1.** Doxorubicin uptake analysis in PTCM.

**Table S1.** List of identified proteins common to both spheroids and 2D cells.
**Table S2.** List of identified ECM‐related proteins in spheroids and 2D cells.

## Data Availability

The data that support the findings of this study are available in the main text and the Supporting Information of this article. The mass spectrometry proteomics data have been deposited to the ProteomeXchange Consortium via the PRIDE partner repository with the dataset identifier PXD061082.
