# Supplementary material for: Enhancing therapeutic efficacy through degradation of endogenous extracellular matrix in primary breast tumor spheroids
Source: FEBS J. 2025 Mar 17;292(13):3494–507. doi: 10.1111/febs.70069 (PMC12220849; doi:10.1111/febs.70069)
Supplement: Supplementary file 1 — Fig. S1. Doxorubicin uptake analysis in PTCM. [file FEBS-292-3494-s002.docx]

**SUPPLEMENTARY FIGURE 1**

**
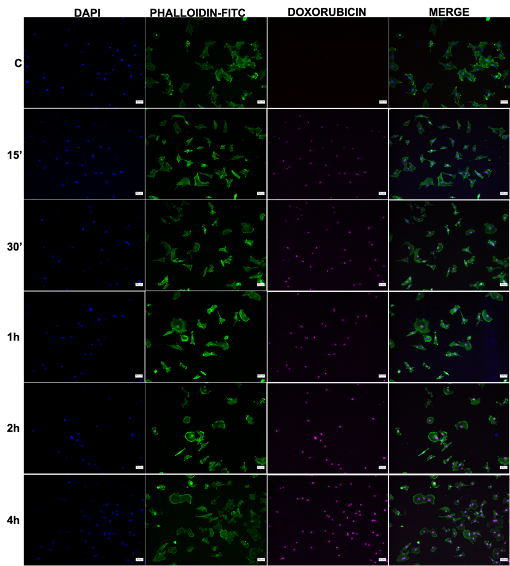
**

**Supplementary Figure 1**

Doxorubicin uptake analysis in primary cells from the tumor mass (PCTM). 20μM Doxorubicin (red) uptake in primary cancer cells, stained with phalloidin-FITC (green) and DAPI (blue) at different times (15’, 30’, 1h, 2h, 4h). Scale bar: 10 μm.
